# Protocol for teen inflammation glutamate emotion research (TIGER): Toward predictors of treatment response and clinical course in depressed adolescents

**DOI:** 10.1016/j.bbih.2023.100718

**Published:** 2023-12-20

**Authors:** Saché M. Coury, Vanessa López, Zia Bajwa, Jordan M. Garcia, Giana I. Teresi, Kate R. Kuhlman, Yan Li, Steve Cole, David J. Miklowitz, Ioannis Pappas, Tiffany C. Ho

**Affiliations:** a Department of Psychology, University of California, Los Angeles, Los Angeles, CA, USA; b Department of Neuroscience, Columbia University, New York, NY, USA; c Department of Psychiatry, Columnia University, New York, NY, USA; d School of Social Work, University of Washington, Seattle, WA, USA; e Department of Psychology, University of Pittsburgh, Pittsburgh, USA; f Department of Psychological Science, School of Social Ecology, University of California Irvine, Irvine, CA, USA; g Cousins Center for Psychoneuroimmunology, Semel Institute for Neuroscience and Human Behavior, University of California Los Angeles, Los Angeles, CA 90095, USA; h Department of Radiology and Biomedical Imaging, University of California, San Francisco, CA, USA; i Departments of Psychiatry and Biobehavioral Sciences and Medicine, Division of Hematology-Oncology, University of California Los Angeles, Los Angeles, CA 90095, USA; j Department of Psychiatry and Biobehavioral Sciences, David Geffen School of Medicine, University of California, Los Angeles, Los Angeles, CA, USA; k Laboratory of NeuroImaging, Stevens Neuroimaging and Informatics Institute, Keck School of Medicine, University of Southern California, Los Angeles, CA, USA

**Keywords:** Depression, Adolescence, Social stress, Fronto-cingulate-limbic circuits, Magnetic resonance spectroscopy, Selective serotonin reuptake inhibitors

## Abstract

Adolescent-onset depression is a prevalent and debilitating condition commonly associated with treatment refractory depression and non-response to first-line antidepressants. There are, however, no objective tests to determine who may or may not respond to antidepressants. As depressed adolescents are especially vulnerable to the lifelong consequences of ineffectively-treated depression, it is critical to identify neurobiological predictors of treatment non-response in this population. Here, we describe the scientific rationale and protocol for the Teen Inflammation Glutamate Emotion Research (TIGER) study, a prospective 18-month investigation of 160 depressed adolescents who will be assessed before and after treatment with selective serotonin reuptake inhibitors. TIGER will be using ultra-high field imaging to test the effects of acute stress and antidepressant treatment on inflammatory and glutamatergic processes hypothesized to underlie depression maintenance. Results from this work will motivate future studies testing alternative therapeutics for depressed adolescents at risk for treatment resistant depression. ClinicalTrials.gov Identifier: NCT05329441.

## 1 Introduction

Depression is highly debilitating and prevalent among adolescents. Adolescent-onset depression, compared to adult-onset depression, has been associated with longer, more severe, and more recurrent depressive episodes which are often unresponsive to treatment (Clayborne et al., 2019; Johnston et al., 2019; Maalouf et al., 2011). In 2020, 4.1 million adolescents, aged 12–17 years old, were estimated to have experienced at least one major depressive episode, with 41.6% of adolescents in the U.S. receiving treatment in the same year (National Institute of Mental Health, 2022). Alarmingly, up to 40% of adolescents with depression do not respond to first-line antidepressant treatment (i.e., selective serotonin reuptake inhibitors [SSRIs]) (Miller and Campo, 2021; Vitiello et al., 2011), herein termed treatment-non response (TNR). TNR is typically defined as an insufficient reduction (<50%) in clinical depressive symptoms after one adequate treatment trial (i.e., therapeutic dose for at least 8 weeks; (Birmaher and Brent, 2007; Nierenberg and DeCecco, 2001). Ineffectively treated depression often results in a cascade of chronic interpersonal and health difficulties throughout the lifespan that are associated with devastating outcomes, including cardiovascular disease and suicide (Dunn and Goodyer, 2006; Naicker et al., 2013).

Thus, there is a clear need to understand why a substantial portion of adolescents with depression do not respond to first-line antidepressant treatment and what neurobiological pathways may be contributing to TNR to SSRIs. Addressing this critical knowledge gap will equip clinicians and scientists with the means to improve response rates, reduce the number of ineffective treatment trials, and lay the foundation for developing more targeted and effective therapeutics that will promote health outcomes in a young and vulnerable population.

Several specific clinical risk factors, such as earlier age of depression onset, exposure to childhood trauma, and a greater number of psychiatric comorbidities, have been associated with TNR to SSRIs in depressed adolescents (Miller and Campo, 2021; Vitiello et al., 2011; Dunn and Goodyer, 2006; Curry et al., 2011; Dwyer et al., 2020; Emslie et al., 2010). However, to date, there are no objective diagnostic tests indicating which depressed adolescents may respond effectively to first-line SSRIs. Furthermore, depressed adolescents who may respond to SSRI treatment initially may still vary in their time to remission; in fact, up to 80% of depressed adolescents who are treated with SSRIs experience recurrence within two years (Vitiello et al., 2011; Dunn and Goodyer, 2006; Curry et al., 2011; Dwyer et al., 2020; Emslie et al., 1997, 2010; Burcusa and Iacono, 2007). Given the clear heterogeneity in response to SSRI treatment—and in clinical course more generally—it is likely that there are distinct neurobiological mechanisms and/or predictors that underlie subgroups of depressed adolescents defined on the basis of their illness trajectories. In this context, understanding the processes involved in the maintenance of depression is critical for identifying the neurobiological mechanisms of TNR and for improving treatment response and clinical outcomes.

Sustained threat to stress—particularly social stressors characterized by interpersonal conflict—has been implicated as a major risk factor for the onset and maintenance of depression in adolescents (Hammen, 2009). The chronic and compounding nature of these stressors, and how they diminish self-worth, may explain why they are more predictive of depressive episodes and symptoms compared to exposure to other life stressors (Kautz et al., 2020; Ojha et al., 2022; Slavich et al., 2020; Vrshek-Schallhorn et al., 2015; Sheets and Craighead, 2014; Stroud et al., 2011). There is now consistent evidence that there are bidirectional associations between inflammation and depressive symptoms among adolescents (Mac Giollabhui et al., 2021; Moriarity et al., 2020), with compelling theoretical models that outline how exposure to social stressors trigger the same neuroimmune pathways that govern the detection and behavioral responses to pathogens and bodily injuries (Miller and Raison, 2016; Slavich and Irwin, 2014). These stress responses include increased inflammatory signaling in the periphery that ultimately impact multiple pathways in the central nervous system. Specifically, pro-inflammatory cytokines facilitate the release of enzymes that constitute the kynurenine pathway, resulting in the production of neurotoxic metabolites such as quinolinic acid, which bind to N-methyl-D-aspartate (NMDA) receptors, stimulating the release of glutamate (Guillemin et al., 2001; Miller, 2013). Additionally, several cytokines, including tumor necrosis factor-alpha (TNF-α), have been shown to directly compromise structural integrity of astrocytes (which play an important role in reuptake of extracellular glutamate), and reduce the expression of glutamate transporters on oligodendrocytes (Haroon et al., 2017). Over time, the neuroexcitoxic effects of excessive glutamate ought to lead to observable alterations (e.g., decreased gray matter morphometry and white matter microstructure) in brain circuits relevant to the detection and regulation of social stress.

More recently, this confluence of inflammation and glutamate from the lens of stress biology has been applied to our understanding of adolescent depression. Specifically, our group has reported evidence that higher levels of inflammation are associated with higher levels of glutamate in the anterior cingulate cortex in depressed adolescents (Ho et al., 2021), and that higher levels of inflammation are also associated with lower white matter connectivity in fronto-cingulate-limbic tracts (i.e., anterior segments of the corpus callosum (Ho et al., 2022a). Prior work has also shown that functional and structural alterations in fronto-cingulate-limbic circuity—including the anterior cingulate cortex, ventromedial prefrontal cortex, amygdala, and hippocampus—are robust neurophenotypes of adolescent depression (Haroon et al., 2017; Connolly et al., 2013, 2017; Ho et al., 2014, 2015, 2017, 2022b; Kaiser et al., 2015; Kerestes et al., 2013; LeWinn et al., 2014; Miller et al., 2015; Mulders et al., 2015). Interestingly, animal models indicate that inflammation may influence these depression-relevant brain circuits (Miller, 2013; Felger, 2018). Nevertheless, it is unclear whether excessive glutamate is a mechanism by which higher levels of stress-related inflammation may contribute to depression maintenance (or recurrence) and the extent to which these processes are predictive of treatment response to SSRIs.

Here, we outline the rationale and protocol for the Teen Inflammation Glutamate Emotion Research (TIGER) study (R01MH127176; NCT05329441), which seeks to address important gaps in our understanding of how glutamate may explain how peripheral inflammation contributes to TNR and longer-term clinical outcomes in depressed adolescents. The current study will be the first in the field to mechanistically examine the roles of inflammation and glutamate on biobehavioral systems related to sustained threat to social stress, and how those processes contribute to TNR and depression trajectories in adolescents. Leveraging ultra-high field imaging at 7T provides the opportunity to also reliably assess other metabolties, such as γ-aminobutyric acid (GABA), in notoriously difficult corticolimbic regions by providing more reliable localization for regions of interest, improved spectral resolution, and more accurate metabolite quantification (Henning, 2018; Ladd et al., 2018; Lally et al., 2016). Our multimodal results are expected to motivate future studies testing alternative therapeutics for depressed adolescents at risk for treatment resistant depression. Below, we summarize information regarding study aims, participant eligibility/inclusion, the study design, and general methodological procedures.

## 2 Study aims & hypotheses

In Aim 1, we will establish whether psychosocial stress increases levels of peripheral inflammation and levels of glutamate in fronto-cingulate-limbic circuits in adolescents with depression.

In Aim 2, we will define TNR based on whether depressed adolescents exhibit at least a 50% reduction in CDRS-R scores after 12 weeks of SSRI treatment. We will compare TNR versus non-TNR groups on TSST-related changes to inflammatory cytokines and glutamate. We will also test whether TSST-related changes in glutamate before and after SSRI treatment mediate the associations between TSST-changes in cytokines at baseline and TNR status (binary outcome). We will also explore models using depression severity as dimensional outcomes instead. We hypothesize that compared to non-responders, responders will show greater stress-induced increases in cytokines and glutamate, as well as stronger positive associations between cytokines and glutamate in threat neurocircuitry.

In Aim 3, we will characterize 18-month trajectories in adolescent depression from behavioral, inflammatory, and neural indicators of threat to social stress. Seven repeated clinical assessments over 18 months will support identifying depression trajectory subgroups (e.g., persistent depression, gradual remission, etc). For binary outcomes (diagnosis of depression), we will use information from clinical interviews to determine remission, relapse, or recurrence status at each timepoint. For dimensional outcomes, we will use symptom severity scores. We hypothesize that increased levels in indicators of sustained threat before and after SSRI treatment, as well as severity of past and ongoing life stress, will predict increased likelihood of experiencing chronic and unremitting depression over 18 months.

For all of the statistical analyses testing these hypotheses, potential demographic (e.g., age, gender), clinical (e.g., age of depression onset, number of comorbidities, presence of childhood trauma), or biological (e.g., percentage of gray matter in MRS voxel) covariates will be included if they are correlated with our outcome variables*.* Statistical significance will be set at α = 0.05. We will apply false discovery rate (FDR) correction as appropriate. In all simulation analyses for estimating sample size based on 1-β = 0.8, effect sizes (*r* or η2 or mean ± SD) were based on preliminary data or cited literature. For the hypotheses in Aims 2 and 3 that require logistic ridge regression analysis, we plan to sample 90% data as the training set and 10% as the testing set across 10 iterations; power was therefore calculated based on 90% of the sample after accounting for attrition (*N* = 108). We will put in rigorous effort to minimize missing data, including scheduling sessions around families’ availability, repeating scans when data were not useable due to movement or scan/user error, and providing bonus incentives for completing all follow-up assessments. For all longitudinal analyses, we will use flexible modeling approaches that allow individuals who provide at least one assessment to be included in our estimation of the effects of interests.

## 3 Methods

### 3.1 Recruitment of target sample

Participants will be recruited through flyers posted in the community and in local clinics, UCLA data registries, and social media advertisements targeting individuals in the general Los Angeles County (including Ventura, Orange County, Riverside). The target sample for this study will be 160 treatment-seeking depressed male and female adolescents of all ethnicities ages 14–21 years old.

### 3.2 Study eligibility

Inclusion criteria includes meeting DSM-V criteria for a depressive disorder, post-pubertal status (Tanner stage ≥3) as determined through self-report Tanner staging (Tanner and Whitehouse, 1976), and starting treatment with either fluoxetine or escitalopram by a clinician. For those under age 18, participants must have a parent/legal guardian who is able to answer questions about family history of mental disorders and the child's behavioral symptoms. All participants and guardian(s) must be able and willing to provide written informed assent and consent, respectively, and able to understand all study procedures (e.g., fluency in English). Exclusion criteria includes a primary Axis I disorder based on DSM-V criteria besides depression (comorbid anxiety diagnosis is allowable), meeting criteria for mania, psychosis, or substance use disorders, stimulant usage, use of antidepressants or other central nervous system medications within five half-lives, a mild concussion within the previous six weeks or severe concussion in the individual's lifetime, evidence of severe psychiatric symptoms requiring hospitalization, first-degree relative with suspected past or present diagnosis of bipolar disorder or schizophrenia, and previous diagnosis of any major illness (e.g., childhood cancer, multiple sclerosis), neurological disorder (e.g., seizures), pervasive developmental disorders, or inflammatory conditions that might interfere with the goals and measures of the study. Any contraindication to MRI scanning (e.g., orthodontics/braces, non-removable piercings, pregnancy), venipuncture (e.g., hematoma, history of trypanophobia, vasovagal reactions to venipuncture), or current SSRI treatment is also exclusionary.

### 3.3 Study design

**Fig. 1:**
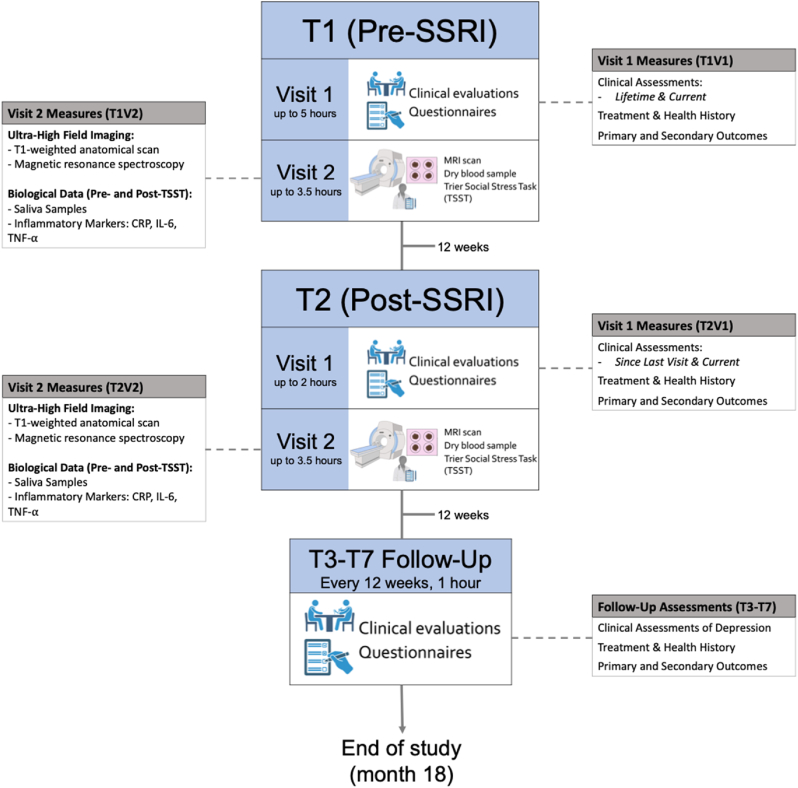
**Study design for TIGER.** TIGER will be an 18-month longitudinal study consisting of seven timepoints, each 12 weeks apart. The first two timepoints (T1, pre-SSRI treatment, and T2, post-SSRI treatment) will consist of two visits each: visit 1 (V1) will comprise clinical evaluations and self-report questionnaires and visit 2 (V2) will consist of multimodal neuroimaging (see Table 1) and biospecimen collection. After T2, participants will complete five follow-up assessments every 12 weeks (T3-T7), which will consist of clinical assessments of depression, updated treatment and health history information, and completion of self-report questionnaires.

160 treatment-seeking depressed adolescents who meet includion criteria will complete study procedures every 3 months for 18 months (7 timepoints total). The first assessments of this 18-month study capture the pre-SSRI period (T1) and after 12 weeks of the SSRI (T2). Both T1 and T2 will consist of two distinct visits (Visit 1, V1, and Visit 2, V2), spaced approximately 1–2 weeks apart (see Fig. 1). At V1, we will administer clinical interviews (and at T1V1 we will determine final study eligibility as we will review exclusionary criteria) and self-report surveys to capture symptomatology and clinical history including treatment information (this is relevant mostly at T1). At V2, we will assess peripheral measures of pro-inflammatory cytokines and glutamate in fronto-cingulate-limbic circuits utilizing ultra-high field imaging with a 7T MR scanner before and after a social stress paradigm. Finally, every 12 weeks thereafter through 18 months (T3 to T7), we will administer brief clinical interviews and self-report surveys in order to collect information on depression symptoms and diagnosis in order to characterize, and identify predictors of, trajectories of depression.

The rationale for the intervals in our study design are as follows: as the vast majority of depressed adolescents seen in the clinics we will be recruiting from will be treated with antidepressants for the first time, we will minimize heterogeneity in antidepressant treatment at study enrollment by only including those who will be initially treated with fluoxetine or escitalopram. The clinical recommendations for treating depressed adolescents with SSRIs are to start with a low dose to minimize potential side effects and then to increase the dose as quickly as tolerated by the patient to reach a therapeutic dose based upon FDA guidelines; if the patient does not respond after 4–6 weeks at a given dose, the recommendation is to gradually increase this dose to the maximum dose approved by FDA guidelines that can be safely tolerated. If the patient does not improve even after being at the FDA-approved maximal dose for at least 6 weeks, the recommendation is to switch to another SSRI. Due to these clinical practice parameters, we anticipate and expect virtually all participants to be on an SSRI (and, specifically, fluoxetine or escitalopram for at least 6 months of the study). However, we also expect and anticipate that doses will be reduced or augmented at any time at the direction of the treating clinician; we will account for these dose changes and differences statistically as covariates. After T2, participants may not necessarily remain on SSRIs and are likely to undergo various changes in treatment, particularly for the TNR group. Regardless, we will collect clinical information regarding history of medication usage at T1 and current medication usage (and psychotherapies) every 12 weeks from both the child and parent throughout the study, including assessment of adherence to medication, switching medications or to other antidepressant classes, and reasons for any medication changes (e.g., side effects, cost). Maintaining consistent intervals between assessments will thereby facilitate longitudinal analyses.

### 3.4 Measures of depression

We will be administering the K-SADS-PL (Kaufman et al., 1997) for assessing Axis I disorders in all study participants (as assessments for depressive disorders do not meaningfully differ between K-SADS-PL and similar instruments designed for adults such as the Structured Clinical Interview for DSM Disorders). The K-SADS-PL will be used at baseline to determine depression diagnosis according to the DSM-5 and study eligibility (as potential participants with a history of mania, psychosis or who have current substance use disorders will be excluded from the study). Depression diagnosis will also be used as an outcome measure to determine remission versus recurrence throughout the study period. We will also administer a modified version of the Family Interview for Genetic Studies (FIGS; Maxwell, 1992) at T1 in order to exclude potentially depressed participants who have first-degree relatives with mania or psychosis. The study group will convene weekly to discuss any ambiguous codes.

To assess depression severity dimensionally, we will administer the Children's Depression Rating Scale-Revised (CDRS-R), a 17-question, clinician-rated scale assessing dimensional scores of depression severity, and the Reynolds Adolescent Depression Scale, 2nd Edition (RADS-2), which is a 30-item self-report questionnaire assessing depression severity for the past two weeks. Both scales have been widely used in clinical trials and research studies of adolescent depression demonstrate sensitivity and reliability in assessing depression symptoms across time (Poznanski et al., 1984; Reynolds, 2010). Additionally, we will administer the Patient Health Questionnaire 9 (PHQ-9), a 9-item screener for depression that is commonly used in clinic and research settings for adults and adolescents and for assessing rapid symptom changes (Kroenke et al., 2001).

### 3.5 Other clinical and self-report measures

To support exploratory hypotheses and collect collateral information on factors that relate to and/or influence depression and inflammation, we will be administering additional instruments and questionnaires. Specifically, to examine anxiety symptoms, we will administer the Multidimensional Anxiety Scale for Children (MASC-2), a 39-item questionnaire assessing anxiety symptoms (March et al., 1997), and the Generalized Anxiety Disorder-7 (GAD-7), a 7-item screener for generalized anxiety disorder that is commonly used in both clinic and resarch settings for adults and adolescents (Spitzer et al., 2006). Suicidal and other self-injurious thoughts and behaviors will be assessed using common instruments in the field for this population, including the Suicidal Ideation Questionnaire (SIQ (Reynolds, 1987)), the Columbia Suicide Severity Rating Scale (C-SSRS; (Posner et al., 2011), and the Self-Injurious Thoughts and Behaviors Interview (SITBI (Nock et al., 2007)). We will also collect information on stress exposure, including current levels of stress using the Perceived Stress Scale (PSS (Cohen et al., 1983)), lifetime exposure to adversity using the Stress and Adversity Inventory for Adolescents (Slavich et al., 2019), experiences of discrimination using the Adolescent Discrimination Distress Index (Fisher et al., 2000), and emotion regulation tendencies using the Difficulties in Emotion Regulation Scale (Gratz and Roemer, 2004).

### 3.6 Trier Social Stress Task (TSST)

A modified version of the Trier Social Stress Task (TSST) will be administered at T1V2 and T2V2. This adapted TSST involves no deception and follows procedures similar to those used by our group in adolescent populations (Ho et al., 2020; Kircanski et al., 2019; Miller et al., 2019). Following the first set of brain imaging acquisitions and the first blood sample collection, participants will be instructed by an experimenter that they will be completing a stress test. There will be two versions of the stress test: a five-minute arithmetic task or a five-minute speech task (regardless of the task, participants will be given 5 minutes of prep time beforehand). Participants will be randomly assigned one of the two stress tests at T1 and will be administered the second stress test at T2 to minimize habituation with repeated testing and ensuring that the order of the two tests are counterbalanced across the sample.

Upon arrival to the session, before, and after the TSST, participants will provide ratings of ten mood states (*Excited, Afraid, Confused, Sad, Energetic, Angry, Happy, Tired, Tense)* based on the Positive and Negative Affect Schedule (PANAS; Crook et al., 1998) on a visual analogue scale from 0 to 10. These ratings will be used as tertiary outcome measures of behavioral responses to social stress. During administration of the TSST, we will also collect saliva samples repeatedly to explore changes in stress hormones before and after an acute stressor. Five saliva samples, each collected approximately 10 minutes apart, will be collected using the SalivaBio Oral Swab Method (Salimetrics, LLC) during the TSST protocol, from which we plan to assay cortisol production. After completing the TSST, participants will complete the remaining scans and provide a secondary blood sample approximately 120 minutes after the first blood sample and then provide a final saliva sample. Additionally, it is possible that secondary stress and anxiety may affect cytokine and CRP levels at Time 1, as this will be the first time most of the participants will have undergone an MRI scan; however, our primary measures of inflammation and glutamate are *within-person differences* before and after the TSST. We will also consider using the PANAS ratings of mood states as covariates in sensitivity analyses.

### 3.7 Inflammatory markers

To acquire inflammatory cytokine information, we will implement a dried blood spot (DBS) protocol at both T1 and T2 using microfluid devices that will permit storage of up to 70 μL at each timepoint (280 μL by the end of T2V2). Prior to arriving at the scanner, participants will be instructed to drink as much water as they can to ensure they are properly hydrated; we will also be providing heat pads and blankets to facilitate blood flow. Although unpublished data from our consultant on the grant, Dr. Andrew Miller, has not indicated significant effects of circadian variation on TNF-α in adults with depression, we will use time since wakening and time of sample collection (as well as BMI) as covariates in sensitivity analyses. Samples will be placed in Ziplock bags with a desiccant for storage in a −80 °C freezer. All DBS samples will be assayed for IL-6, TNF-α, and CRP. All extraction and analyses of blood samples will take place at the Institute for Interdisciplinary Salivary Bioscience at the University of California, Irvine (https://iisbr.uci.edu/) in collaboration with co-investigator, Dr. Kate Kuhlman. All assays will be tested, in duplicate, using commercially available enzyme-linked immunoassay kits (e.g., IBL International, Hamburg, Germany) following the manufacturers’ protocol without modification. Optical density will be determined at 450 nm using a microplate reader and inflammatory protein concentrations quantified against a five-parameter logistic curve fit generated standard curve. Detection ranges for IL-6, TNF-α, and CRP are 0.08–5.0 pg/mL, 0.31–20.0 pg/mL, and 0.4–10.0 μg/mL, respectively. Intra-assay coefficients of variability range across populations and collection methods, but for past studies using samples collected via DBS, intra-assay coefficients of variability are expected to be <10%. Samples tested outside the detection range for the assay will be retested at a higher dilution to confirm the result.

### 3.8 Ultra-high field imaging

All MRI acquisitions will be performed on the Siemens MAGNETOM Terra 7T whole body scanner (Siemens Healthcare GmbH, Erlangen, Germany) utilizing ultra-high-field technology at the Center for Image Acquisition (CIA) at the University of Southern California. Below is a summary of each sequence type used in this project. See also Table 1 for a summary of the scan parameters.

**Table 1:** Summary of scan acquisitions and parameters.

Scan Acquistion	Voxel Size (mm^3^)	# of Averages (NEX)	# of Slices (orientation)	FOV (cm)	TR/TE/TI (ms)	Flip Angle (°)	Phase Encoding Direction
**3D T1-weighted MRI**	1.1 × 1.1 × 1.0	1	192 (sagittal)	25.6	4300/1.84/840(inv1)/2370(inv2)	5.0(inv1)/6.0(inv2)	A/P
**rACC sLaser** **SVS MRS**	15 × 15 × 15	64	1 (axial)	NA	4000/29/NA	90	A/P
**dACC sLaser SVS MRS**	15 × 15 × 15	64	1 (axial)	NA	4000/29/NA	90	A/P
**L & R Hippocampus sLaser SVS MRS**	15 × 15 × 15	128	1 (axial)	NA	4000/29/NA	90	A/P

### 3.9 T1-weighted anatomical MRI

A high-resolution 3D T1-weighted anatomical image will be acquired at both visits using a magnetization-prepared 2 rapid gradient echo (MP2RAGE) sequence (5 min 20 sec), with parameters tuned to optimize tissue contrast between gray and white matter at 7T. We will utilize the high-resolution images from this acquisition to prescribe more structurally accurate voxels in the MRS acquisition, implement structural processing pipelines, and to facilitate brain atlas registration of other image acquisitions in the processing pipeline.

### 3.10 Magnetic resonance spectroscopy (MRS)

To characterize neurometabolic data, we will implement single-voxel (SV) magnetic resonance spectroscopy (MRS) imaging before and after the TSST. We will utilize the sLASER sequence for metabolite detection *in vivo*. A custom-made developed excitation pulse (EB 2000E, 4 ms) will be used for X slice selection and four refocusing pulses will be used for Y and Z slice localization (Kaiser et al., 2022) and VAPOR water suppression will be used. Optimization of transmit gain and higher order shimming will be performed prior to MRS acquisitions for each voxel of interest, including the rostral anterior cingulate cortex, dorsal anterior cingulate cortex, and the left and right hippocampus.

All metabolite concentrations will be estimated using fitting in LCModel (Provencher, 2009) and expressed as ratios relative to total creatine (i.e., creatine and phosphocreatine) as these levels are relatively high and stable across different tissue types in the brain and, thus, often used as an internal reference standard. Only voxels that have relative Cramer-Rao lower bounds (CRLBs) < 25% for the metabolite of interest will be considered in further analysis. Finally, the effects of fluid within each voxel will be corrected by dividing the metabolite concentrations by the fraction of non-fluid components (i.e., WM and GM). Given the dynamic cycling between glutamine and glutamate (extracellular glutamate is converted into glutamine in astrocytes before being released and taken up by neurons where it is synthesized into either glutamate or GABA), we will also consider the total sum of glutamate and glutamine (Glx), and the ratio of glutamine to glutamate, as complementary indices of glutamate metabolism (Brennan et al., 2017; Cooper et al., 2021).

## 4 Follow-up assessments

Upon completion of T1 and T2, participants will complete five follow-up assessments every 12 weeks (T3-T7), which will consist of clinical assessments of depression, updated treatment and health history information, and completion of several self-report questionnaires, including our primary and secondary outcomes: RADS-2, MASC-2, PHQ-9, GAD-7, and PSS.

## 5 Discussion

This study will be the first to investigate the roles of inflammation and glutamate on biobehavioral systems underlying sustained threat to social stress, and how these processes contribute to treatment non-response (TNR) to SSRIs and clinical course in adolescents with depression. The results from the first aim of the study will represent the largest multimodal investigation characterizing sustained threat to social stress in this population. We will have the opportunity to measure stress-evoked changes in peripheral inflammation and glutamate in fronto-cingulate-limbic circuitry and the extent to which these indicators of sustainted threat to social stress correlate with functional activation patterns in brain regions associated with social rejection.

Results from the second aim of this project will be the first to uncover neurobiological predictors of treatment non-response to SSRIs, which may inform treatment guidelines in this population, and provide preliminary evidence that exaggerated glutamate or altered glutamatergic metabolism may be a mechanistic target for mitigating the effects of stress-induced inflammation on the developing adolescent brain. Finally, the findings from the third aim of this study will be critical for characterizing heterogenous trajectories of clinical course in depressed adolescents beyond initial response to antidepressant treatment and will enable us to identify the earliest points of maximal divergence between trajectory groups. As such, this study will build significantly from seminal studies in the field that have focused on isolated predictors of treatment non-response in adolescents (Dwyer et al., 2020; Asarnow et al., 2009). The use of ultra-high field imaging will afford us unprecedented granularity in distinguishing glutamate from other metabolites with overlapping resonances (e.g., glutamine) compared to acquisitions at 3T or 1.5T, which have lower contrast, signal-to-noise, and spatial resolution compared to MR imaging at 7T (Moriguchi et al., 2019).

One challenge we anticipate is that we will have an unknown distribution of patients who will be TNR at T2. For our power analyses, we have estimated that 40% of the sample will be considered TNR after 12 weeks of SSRIs based on prior clinical trials. Furthermore, due to clinical practice paramters, FDA guidelines, and patient response, we also expect and anticipate that SSRI doses will be reduced or augmented at any time during the study at the direction of the treating clinician. Regardless, we will collect clinical information regarding history of medication usage, current medication usage (including psychotherapies) every 3 months throughout the study, as well as assess adherence to medication, switching medications or to other antidepressant classes, and reasons for any medication changes (e.g., side effects, cost). We will account for these dose changes and differences statistically as covariates in our analyses. Also, we will acquire data on depression symptom severity (i.e., CDRS-R, PHQ-9) and will be able to use a *dimensional* measure of depression symptom changes as an outcome in our statistical models. Another potential challenge we will face involves the timing of biospecimen collection: we aim to collect the post-TSST blood sample to measure inflammatory markers 120 minutes post stress-onset to capture acute stress responses, but we may be missing stress reactivity windows for some participants by only obtaining one post-stress sample. We will use time of collection as a covariate for this study but future work may benefit from the collection of multiple post-stress blood samples. Finally, this study employs stress induction through the TSST, but future work should also mechanistically investigate stress *reduction* paradigms to examine how lowering stress may acutely impact peripheral inflammation and glutamate metabolism in depressed adolescents.

As the overarching goal of this work is to characterize an inflammation-related endophenotype of treatment-resistant depression among youth, it is important to emphasize that we are using SSRIs as a mechanistic probe to determine TNR status in our sample of depressed adolescents. However, a secondary consequence of our study design is that we will also be able to explore other questions, such as whether SSRIs generally reduce peripheral inflammation and/or affect glutamate regardless of TNR status and also if there are interesting patterns regarding the distribution of distinct specifiers or subtypes of depression present in our sample despite all participants being recommended either fluoxetine or escitalopram by their own provider. Regardless of the outcome of results, our findings will be important for sharpening future directions of this work, including supporting or refuting our argument that stress mechanisms account for treatment-resistant depression.

In summary, the overall goals of this project are to elucidate the neurobiological mechanisms by which sustained threat to social stressors contribute to treatment resistant depression and how these mechanisms may influence longer term symptom trajectories in depressed adolescents. The findings from this investigation may inform the development of novel and more effective interventions for adolescent-onset depression and for treatment-resistant depression more broadly.

## CRediT authorship contribution statement

**Saché M. Coury:** Writing – original draft, Writing – review & editing, Visualization, Conceptualization. **Vanessa López:** Visualization, Writing – review & editing. **Zia Bajwa:** Writing – review & editing. **Jordan M. Garcia:** Writing – review & editing. **Giana I. Teresi:** Conceptualization, Writing – review & editing. **Kate R. Kuhlman:** Conceptualization, Writing – review & editing. **Yan Li:** Conceptualization, Writing – review & editing. **Steve Cole:** Conceptualization, Writing – review & editing. **David J. Miklowitz:** Conceptualization, Writing – review & editing. **Ioannis Pappas:** Conceptualization, Writing – review & editing. **Tiffany C. Ho:** Conceptualization, Funding acquisition, Project administration, Supervision, Writing – original draft, Writing – review & editing.

## Declaration of competing interest

The authors declare the following financial interests/personal relationships which may be considered as potential competing interests:

Dr. Miklowitz receives research support from the National Institute of Mental Health, the Baszucki Brain Research Fund/Milken Foundation, Danny Alberts Foundation, Attias Family Foundation, Carl and Roberta Deutsch Foundation, and Max Gray Fund; and book royalties from Guilford Press and John Wiley and Sons.

All other authors report no biomedical conflicts of interest.

Tiffany C. Ho reports financial support was provided by National Institute of Mental Health. Saché M. Coury reports financial support was provided by National Institute of Mental Health. Steve Cole reports financial support was provided by National Institute of Mental Health. David J. Miklowitz reports financial support was provided by National Institute of Mental Health. Kate R. Kuhlman reports financial support was provided by National Institute of Mental Health. Yan Li reports financial support was provided by National Institute of Mental Health. Ioannis Pappas reports financial support was provided by National Institute of Mental Health. David J. Miklowitz reports a relationship with Guilford Press that includes: employment. David J. Miklowitz reports a relationship with John Wiley & Sons Inc that includes: employment. Author research support from the Baszucki Brain Research Fund/Milken Foundation - D.J.M. Author research support from the Danny Alberts Foundation - D.J.M. Author research support from the Attias Family Foundation - D.J.M. Author research support from the Cal and Roberta Deutsch Foundation - D.J.M. Author research support from the Max Gray Fund - D.J.M.

## Data Availability

No data was used for the research described in the article.
